# The Institutional Worker

**Published:** 1918-07-13

**Authors:** 


THE
### INSTITUTIONAL WORKER
Being a Special Supplement to "The Hospital"

### OUR BUREAU OF INFORMATION.

### Rules for Correspondents.

1. Every letter must be accompanied by the coupon to be cut from the back cover (inside page) of THE HOSPITAL, current issue, and must contain the name and address of the correspondent with pseudonym for publication if desired. Replies by post cannot be given save under exceptional circumstances at the Editor's discretion.
2. Letters from Approved Homes in reply to special needs published in the Bureau should state terms and full particulars, and be sent prepaid under cover to the Editor of the Bureau with name written across coupon for identification.
3. Proprietors of Homes which have not yet been entered on the List of Approved Homes, but have spare accommodation likely to suit special needs, are invited to write for an application form for registration. The fee for registration, which includes two announcements of the Home in the Bureau and other privileges, is 10s.
4. All communications to be addressed to the Editor of THE HOSPITAL, 28 Southampton Street, Strand, London, W.C. 2, and marked " Bureau of Information."

### INSTITUTION FACTS AND FIGURES.

### The Preservation of Fruit and Vegetables.

THE threatened scarcity of jam during the coming winter, and the poor fruit crops this summer, are causing the housekeeper considerable anxiety. The public have settled down to what was thought at one time was a scanty allowance of sugar, and the careful housewife has even been able to save out of it with a view to jam-making. But now the fruit is the difficulty.

Lucky are the residents in institutions with their own orchard and garden. As we showed last year, the matrons of these establishments were able to stock their store-cupboards with jam and bottled and preserved fruits, curry, and other good things to tide them over many months. They should, therefore, be interested in an article on the Preservation of Fruit, by Mr. Herbert E. Durham, Sc.D., which recently appeared in the *British Medical Journal*, from which we take the following information :—

#### BOTTLING FRUIT WITHOUT SUGAR.

Fruit bottled in plain water becomes more or less tasteless, for the reason that a diffusion of the sugar and other components in the fruit takes place from within it into the water until an equilibrium is established. This may be avoided by making use of juice from some of the fruit for filling the bottles. The juice may be made either by cooking some of the fruit in a little water and straining off the juice through a cloth or by the use of a fruit press, of which there are several patterns on the market. The latter process is more economical, and saves time, for leaves and stalks need not be separated from the fruit. The juice thus obtained should be heated before it is put into the bottles. The same sort of juice need not necessarily be used—for instance, red-currant juice may be used for raspberries, and *vice versa*.

#### FRUIT PASTE.

A useful method of conserving fruit is to convert it into a paste. This paste can be utilised for making sauces to eat with puddings, to work up into jam, and so on. Whilst it can best be prepared by the use of sugar, more especially in the case of apples, a product of good keeping quality can be made without it. For example: Apples should be cut up or waste cores and skins need only be used; they should be cooked with the smallest quantity of water possible. When soft they should be allowed to cool, when they will be ready to pass through a fine cloth or sieve to separate pips, etc. The pulp should then be slowly reduced by heat. To prevent burning, it should be continually stirred. It is a good plan to interpose a piece of wire gauze or sheet iron between the flame and the saucepan. Reduction may be regarded as complete when a wooden spoon stands stiffly alone in the pulp. The pulp may be then placed in well-greased baking-trays in layers from a quarter to half an inch thick. It should be then left to dry on a rack over the range, in a slow oven, a hot cupboard, or bright sunlight until the residue is fairly hard but still plastic. It may then be rolled up or cut into squares and stored in paper bags in a dry cupboard. If sugar be used in this preparation, a tenth to a fifth of the weight of pulp may be added.

Black-currants, raspberries, etc., can be made into a paste by the methods already described. It is, however, preferable to pass them two or three times through a press in order to avoid loss of juice.

Apple or quince paste may be made into jam or purée by adding about five times the weight of water, warming slowly for twenty minutes, then just raising to the boil and stirring for a few seconds.

#### TOMATO PASTE.

Boil the tomatoes carefully in water without breaking the skins. Drain the water off as completely as possible, remove the pips and skins—this can be done by straining through muslin or passing the fruit through a press; add a little salt and, if desired, cayenne and allspice to the pulp. Reduce slowly as indicated in the process for apples, pour into trays, dry, and roll up. This mass keeps perfectly. Small pieces mixed with water may be used to flavour soups, stewed haricots, or spaghetti.

#### DRIED FRUIT.

The fruit may be dried without any special outfit. On a small scale a fair quantity can be done with an ordinary baking oven. In a house which has a linen or airing cupboard containing a hot-water cylinder, the process can be carried out on a large scale. When dried hard they keep perfectly in paper bags. Preliminary treatment in a caustic-soda bath or by sulphuring is quite unnecessary. The fruit should be a good size, perfect, and nearly, if not quite, ripe. Fruit which is not perfectly ripe may be spread out on trays made of gauze, wicker, or slats of wood, and placed on the rack over the kitchen range for a few days to complete ripening. This in any case is desirable, for it helps to procure full flavour. Three or four heatings in a cool oven are required. For the primary heating the temperature should not exceed 115° to 120° F.; for the second or third heatings 150° F. is the maximum temperature and 160° for the final heating. Too high a temperature leads to rupture of the skin and escape of the juice. Each heating takes about twenty-four hours, at the end of which time the fruit should be allowed to cool. The fruit should be turned during the period. To avoid the risk of overheating, the oven door must be left open. If the hot cupboard is made use of for drying there is little or no risk of overheating. Treatment for a week every day or every few days, with daily withdrawal to cool, is necessary to complete drying. With complete desiccation, followed by storage in a dry place, perfect conservation is obtained.

#### DRIED VEGETABLES.

Potatoes dry very rapidly in slices about a quarter of an inch thick. Young broad beans and green beans also dry readily. Preliminary scalding in boiling water for five seconds is not necessary.

### JULY QUESTIONS.

### 1. War Vacancies.

**Give model "Terms of Appointment" applicable for the substitute of a hospital's administrative official who has been called to the Colours. Provision to be made for the possible return to duty of the official previous to the termination of the war and the filling of the appointment in case of the non-return of the official concerned. Describe fully any experiences in this connection that may prove of value to hospital managers generally.**

### 2. Bedroom Suppers for Nurses Off Duty.

**In those hospitals having no kitchen in the Nurses' Home what arrangements can be made whereby the sisters and nurses who are off duty in the evening may have the comfort of supper in their bedrooms? What method may be adopted in order to avoid the dining-room maid being faced by a more or less depleted pantry when she wishes to lay breakfast? (The difficulty seems to be not so much as regards the food as the crockery.)**

#### RULES.

The following rules must be observed:—

1. Contributions must be written on one side of the paper. Brevity and terseness are desirable features. The MSS. must bear the name and address of the sender and be accompanied by coupon to be cut from the back cover (inside page) of the current issue of THE HOSPITAL. A pseudonym must be chosen if the name is not to be published.
2. Contributions must be addressed to the Editor of THE HOSPITAL, Institutional Worker Supplement, 28 & 29 Southampton Street, Strand, London, W.C. 2, should reach him before the end of the current month, and be marked in the left-hand corner "Facts and Figures."

**A minimum payment of Five Shillings will be made for each published answer.**

### QUESTIONS INVITED.

IN connection with our Question Box, we offer every month five shillings each for the two best questions which are sent in for consideration. The questions may be on any institutional subject and concern any department, from the secretary's to the porter's, or the matron's to the domestic staff; the one essential is that they must be practical and deal with points that have a definite relation to hospital or institutional administration, upkeep, finance, or management. Points relating to artificers' work, housekeeping, the laundry, out-patients, and other practical matters will be welcome. It is hoped that thus, when difficult or doubtful points arise in the course of their work, institutional workers will be encouraged to put them in the form of a question and send them to the Editor, THE HOSPITAL Bureau of Information, 28 & 29 Southampton Street, Strand, W.C. 2, marked "Question Box."

The best questions will be published in due course and our readers will be given the opportunity of answering them. Thus all institutional workers may be able to co-operate with the view of helping the work and smoothing the difficulties of each other. In short, we want the Question Box of the *Institutional Worker* to be freely used by every worker habitually.

### ENQUIRIES AND ANSWERS.

### *MISCELLANEOUS.*

### Fire Drills.

WE are of opinion that a monthly drill would be sufficient. As many of the nursing and domestic staff as can be released for a few minutes should attend it. Those who are not present on one occasion should have an opportunity of attending on the next; a record of attendances should therefore be kept. As a relatively small number only would be required to handle apparatus, a fire picket should be appointed each month. This will avoid confusion. The picket should be composed of half day and half night staff. The men-servants should be permanent members of the picket and should have their duties assigned.—MATRON.

### Whirlpool Baths.

MESSRS. SHANKS AND CO., LTD., of 81 New Bond Street, London, W., are the makers of whirlpool baths in this country. No; the method of working these baths is not difficult; on the other hand, it is comparatively simple. Before the part to be treated is immersed, the bath is filled with water at about 100° F., and the temperature raised to the limit of endurance, which at the highest would not exceed 120°. The "whirlpool" effect is controlled by the turbine, and the movement of the water may be graduated by a simple regulating device. Provision is made for reversing the motion as well as for aëration of the water.—OLIVE.

### Dispensing without Glycerine.

APPLY to the Pharmaceutical Press, 17 Bloomsbury Square, W.C. 1, for a copy of the Addendum to the British Pharmaceutical Codex, 1911, which was published some little time ago. The information given in this publication should help you to overcome any serious difficulty in dispensing owing to the shortage of glycerine and sugar. The price is 1s. net.—HORNET.

### Public Health Service of Midwifery.

THE Women's Co-operative Guild at Hampstead advocates a Public Health Service of Midwifery. Write to Miss Llewellyn Davies, from whom you can obtain an excellent pamphlet upon the subject. Her address is 28 Church Row, Hampstead, N.W.; the price of the pamphlet 3d.—MIDWIFE.

### Manufacturers of Cystoscopes.

MESSRS. ALLEN AND HANBURYS, LTD., of 48 Wigmore Street, Cavendish Square, London, W. 1, are manufacturers of cystoscopes, both the plain examination models and the irrigation and double catheterising instruments.—H. S.

### Incorporated Society of Chiropodists.

WRITE to the Secretary, Incorporated Society of Chiropodists, 1 Silver Street, Bury Street, Bloomsbury, W.C., who will give you full particulars of membership.—CHIROPODIST.

### Address Wanted.

THE address of the Incorporated Society of Trained Masseuses is 157 Great Portland Street, London, W. A coupon should be enclosed with each inquiry. We do not send postal replies.—F. F.

### *EMPLOYMENT AND TRAINING.*

### Training at Thirty-five.

YES, there are a few hospitals which receive candidates for training in general nursing at the age of thirty-five: The Coventry and Warwickshire Hospital, Coventry; Royal South Hants and Southampton Hospital, Southampton; Sheffield Royal Hospital, Sheffield; the Royal Berkshire Hospital, Reading; and Nottingham General Hospital. Obtain "How to Become a Nurse," published by The Scientific Press, Ltd., 28 and 29 Southampton Street, Strand, W.C. 2, price 2s. 10d. post free.—UNITY.

### School of Cookery for Creche Matron.

THE National Training School of Cookery, in conjunction with the Notting Hill Day Nursery, arranges for the training of crèche matrons in the art of cooking. Apply to the Principal of the National Training School, 72, 78 Buckingham Palace Road, London, S.W.—P. A. S.

### Training for Assistant in Radiography.

YOU can obtain a six months' course of instruction in radiography, actino-therapeutics, and medical electricity at Guy's Hospital. Vacancies occur every three months. Apply for further particulars to the Matron, Guy's Hospital, S.E. 1.—TONY.

### Appointment of Health Visitor.

YOUR general and midwifery training should be a sufficient qualification to secure you an appointment as health visitor. The diploma of the Royal Sanitary Institute is not necessarily essential, though it is a useful qualification to hold, especially having regard to the excellent appointments which are open to women in the Public Health Departments throughout the country.—NURSE A.

### *WAR MATTERS.*

### Laboratory Assistants in France.

WRITE to the Secretary, V.A.D. Headquarters, Devonshire House, Piccadilly, S.W. 1, in regard to a post of laboratory assistant in France. As you have much experience in this work you may possibly be successful in obtaining a post such as you require. The rate of pay is 39s. 6d. per week, from which 14s. is deducted for board and lodging.—LYNETTE.

